# Incidence of Complications in Proximal Femoral Nailing for Comminuted Intertrochanteric Femur Fractures in a Rural Population: A Retrospective Analysis

**DOI:** 10.7759/cureus.104187

**Published:** 2026-02-24

**Authors:** Madhu Kumar, Thulasi Raman, Rajesh Rajavelu

**Affiliations:** 1 Department of Orthopaedics, Meenakshi Medical College Hospital and Research Institute, Kanchipuram, IND

**Keywords:** ao/ota classification, mechanical complications, modified harris hip score, proximal femoral nailing, unstable intertrochanteric fractures

## Abstract

Background: Unstable intertrochanteric fractures (AO Foundation/Orthopaedic Trauma Association (AO/OTA) 31-A2 and 31-A3) are technically demanding. In rural care pathways, delays to presentation and surgery, limited implant/operating resources, and restricted access to structured rehabilitation and follow-up may increase the risk of mechanical failure, wound complications, and delayed functional recovery.

Methods: We retrospectively screened consecutive adults treated with proximal femoral nailing (PFN) for intertrochanteric femur fractures between January 2024 and December 2025. Stable AO/OTA 31-A1 patterns were excluded by design; only unstable AO/OTA 31-A2 and 31-A3 fractures with ≥6 months follow-up were included. Data were extracted from records and radiographs. The primary outcome was the Modified Harris Hip Score (MHHS) at final follow-up, and the secondary outcomes included time to radiological union and postoperative complications.

Results: A total of 128 patients were included, with a mean age of 72.6 ± 9.4 years (55.5% were female, and 67.2% had at least one comorbidity). Fractures were 31-A2 in 78 (60.9%) and 31-A3 in 50 (39.1%). Radiological union was achieved in 120 patients (93.8%), with a mean union time of 12.1 ± 2.9 weeks; delayed union occurred in six (4.7%) and non-union in two (1.6%). Mean follow-up was 15.6 ± 4.9 months. The MHHS at final follow-up was 89.4 ± 8.7 for AO/OTA 31-A2 fractures and 84.8 ± 11.2 for AO/OTA 31-A3 fractures (unadjusted). Excellent or good outcomes were achieved in 103 patients (80.4%). Mechanical complications included Z effect in five (3.9%), screw cutout in seven (5.5%), and varus collapse greater than 5° in nine (7.0%). Surgical site infection occurred in eight (6.3%), and limb length discrepancy greater than 1 cm in 10 (7.8%). Outcomes and complication rates were less favorable in 31-A3 fractures compared with 31-A2 fractures.

Conclusion: PFN achieved high union rates and satisfactory functional outcomes in unstable intertrochanteric fractures treated in a rural setting, although mechanical complications and wound issues were observed, particularly in AO/OTA 31-A3 patterns.

## Introduction

Intertrochanteric fractures remain a major driver of hip fracture morbidity, particularly when instability reflects posteromedial comminution, lateral femoral wall compromise, and reverse-oblique or multifragmentary patterns classified as AO Foundation/Orthopaedic Trauma Association (AO/OTA) classification for AO/OTA 31-A2 and 31-A3 unstable fracture patterns. In such fractures, fixation must restore alignment, preserve offset, and enable early mobilization, yet loss of medial support and high head-neck stresses increase susceptibility to varus collapse, implant migration, and reoperation. Cephalomedullary devices are widely favored because intramedullary load-sharing reduces bending moments and soft-tissue disruption, although complication mechanisms remain clinically relevant [1].

Clinical series consistently report high union rates after proximal femoral nailing (PFN), including in unstable patterns, with predominantly good-to-excellent short-term function, but non-trivial procedure-related complications persist. Mechanical and functional sequelae, such as Z-effect or reverse Z-effect, varus collapse, screw cutout, back-out, shortening, stiffness, and symptomatic lateral thigh pain, are repeatedly documented, occasionally necessitating implant removal or revision [2-4]. Rare iatrogenic events also warrant vigilance; vascular injury related to distal interlocking may present with thigh swelling, pain, and unexplained anemia, requiring prompt imaging when recovery deviates from expectations [5].

Comparative evidence suggests that intramedullary nailing often confers perioperative and early rehabilitation advantages over dynamic hip screw constructs, including shorter operative time, lower blood loss, shorter hospital stay, and earlier progression to weight-bearing, while longer-term hip scores may converge [6]. For unstable fracture biology, implant design may influence mechanical reliability. Pooled analyses generally show similar functional outcomes between proximal femoral nail antirotation (PFNA) and intertrochanteric antegrade nail (InterTAN) across several endpoints, yet signals toward fewer implant failures and reoperations with InterTAN have been reported, especially for screw migration, cutout, and varus collapse [7]. Within AO/OTA 31-A3 patterns, device configuration and nail length have also been linked to differential cutout risk in retrospective cohorts, although confounding by selection and surgeon experience remains a concern [8].

Across devices, modifiable determinants of failure repeatedly converge on reduction quality and implant positioning. Severe osteoporosis has been associated with poorer postoperative function, highlighting the interaction between bone quality, construct mechanics, and rehabilitation potential [9]. Radiographic studies emphasize unsatisfactory reduction as a dominant predictor of cutout, with positioning metrics such as tip-apex distance contributing in some cohorts but not uniformly across all implants and analytic approaches [10]. Morphology-driven considerations further include lateral wall behavior and calcar support; subclassification frameworks have been associated with differences in reduction quality, calcar gapping, and excessive sliding after cephalomedullary fixation, reinforcing the need for careful preoperative characterization and tailored intraoperative strategy [11]. Importantly, radiographic union does not guarantee full recovery, as gait analyses after the PFN demonstrate persistent deficits versus healthy controls at six months, implying ongoing functional vulnerability even after apparent healing [12].

Treatment selection remains context dependent, since arthroplasty can facilitate earlier weight-bearing and faster short-term gains in very elderly unstable fractures, but at the cost of higher blood loss and transfusion needs, with many outcomes converging over time [7,11]. In rural fracture pathways, complication risk is influenced most directly by (i) delayed presentation and time-to-surgery; (ii) constraints in implant availability, imaging, theatre time, and revision capacity; and (iii) limited access to supervised rehabilitation and reliable follow-up. These factors can magnify the functional consequences of mechanical failure and infection, and they also affect the detection and management of complications.

Because this study is a single-implant rural cohort rather than an implant-comparison trial, its contribution is not superiority testing; it provides rural pathway-specific benchmarks for union, complication burden, and morphology-stratified recovery after PFN in unstable AO/OTA 31-A2 and 31-A3 patterns, which are valuable where follow-up continuity and revision options differ from high-resource referral systems. Against this backdrop, the present study aimed to assess complication rates and functional outcomes following PFN fixation of unstable intertrochanteric femur fractures in a rural cohort, with the objective of informing clinical decision-making in similar healthcare settings. By quantifying real-world perioperative events, implant-related complications, and functional recovery after PFN in a rural context, this work seeks to contextualize established technical risk factors and expected outcomes within the practical constraints that shape fracture care outside high-resource referral settings.

## Materials and methods

Study design and setting

This retrospective observational study was conducted at a rural tertiary care teaching hospital after approval from the Institutional Ethics Committee (IEC approval number: MMCHRI/IEC/PG/70/OCT/23). To align with the study aim of estimating complication rates and functional outcomes after PFN in unstable intertrochanteric fractures within a rural cohort, adult patients with unstable comminuted intertrochanteric femur fractures who underwent PFN were identified from January 2024 to December 2025. The January 2024-December 2025 interval was selected to reflect a consistent institutional PFN protocol and documentation system and to ensure at least six months' follow-up for all included patients at the time of data extraction. During this period, core operative principles (reduction goals, implant selection strategy, antibiotic and thromboprophylaxis protocols, and weight-bearing guidance) remained unchanged; any deviations (implant model changes, surgeon turnover, protocol updates) are specified. The study adhered to institutional ethical standards and the principles of the Declaration of Helsinki.

Patient selection and eligibility criteria

Adults aged 18 years or older with radiologically confirmed intertrochanteric femur fractures treated with PFN were screened. Fractures were classified using the AO and OTA classification system, and only unstable comminuted patterns AO and OTA 31-A2 and 31-A3 were included to maintain direct alignment with the target fracture subset in the aim. Exclusion criteria were pathological fractures, polytrauma with ipsilateral lower limb fractures, previous surgery on the affected hip, isolated subtrochanteric fractures without intertrochanteric extension, incomplete clinical or radiographic records, and loss to follow-up before a minimum follow-up duration of six months. Preoperative radiographs were independently classified as AO/OTA 31-A2 or 31-A3 by two reviewers blinded to outcomes. Disagreements were resolved by consensus with a senior trauma consultant. Interobserver agreement was quantified using Cohen's kappa.

Surgical technique

All procedures were performed by experienced orthopaedic surgeons (having more than 15 years of experience) using a standard proximal femoral nail system. Anesthesia was spinal or general, based on anesthetic evaluation and patient comorbidity profile. Patients were positioned supine on a fracture table. Closed reduction was attempted under fluoroscopic guidance, and adequacy was confirmed on anteroposterior and lateral views prior to nail insertion. Nail length and neck shaft angle were selected according to patient anatomy and fracture morphology using a standardized approach. Lag screw placement was optimized with the intended target of central inferior placement on the anteroposterior view and central placement on the lateral view to reduce mechanical failure risk. Open reduction was performed only when an acceptable closed reduction could not be achieved. Intraoperative technical variations were not analyzed because the focus was on postoperative complication burden and functional recovery.

Postoperative management and rehabilitation

Postoperatively, patients received intravenous antibiotic prophylaxis for 24-48 hours and thromboprophylaxis as per institutional protocol. Early mobilization was encouraged with bedside sitting ankle pump exercises and quadriceps strengthening initiated as tolerated. Partial weight bearing with walker support was generally permitted once pain was controlled, and progression to full weight bearing was guided by radiographic stability and evidence of union as determined by the treating surgeon. A structured physiotherapy program was delivered during hospitalization and continued after discharge through outpatient follow-up or local rehabilitation services consistent with rural care pathways.

Follow-up protocol and radiographic assessment

Follow-up visits were scheduled at six weeks, three months, and six months with subsequent visits as clinically indicated. Immediate postoperative AP and lateral radiographs were used to measure tip-apex distance (TAD) with magnification correction, and calcar-referenced TAD (CalTAD). For dual-screw constructs, TAD/CalTAD were recorded for the inferior (lag) screw. Screw position was additionally categorized by femoral head zone (e.g., central-inferior target on AP and central on lateral). Reduction quality was graded as good/acceptable/poor using Baumgaertner alignment and displacement criteria. Measurements were performed by two assessors blinded to outcomes; interobserver reliability for continuous measures was assessed with intraclass correlation coefficients.

Outcome measures and complication definitions

The primary outcome was functional outcome at final follow-up, assessed using the Modified Harris Hip Score (MHHS) [13]. Secondary outcomes were time to radiological union and incidence of postoperative complications. Complications were categorized as mechanical implant failure, screw cutout, varus collapse, Z effect or reverse Z effect, biological delayed union, non-union, superficial or deep infection, and systemic complications documented in inpatient and follow-up records. Delayed union was defined as the absence of progressive radiological healing beyond six months, and non-union as failure of union at nine months with no radiographic signs of healing. Complications were identified from inpatient notes, operative records, and follow-up documentation and were confirmed by a structured review of serial radiographs for mechanical events (cutout, varus collapse, Z-effect). Where possible, complications were adjudicated by two reviewers; disagreements were resolved by consensus.

Data handling and statistical analysis

Data were extracted from case sheets, operative notes, radiology records, and follow-up charts into a structured database capturing demographics, fracture classification, perioperative details, complications, and MHHS outcomes. Records were cross-checked for internal consistency by reconciling operative documentation with radiographic and follow-up entries, and cases with insufficient documentation were excluded based on prespecified criteria. Statistical analysis was performed using Statistical Product and Service Solutions (SPSS, version 29.0; IBM SPSS Statistics for Windows, Armonk, NY). Exploratory subgroup comparisons between prespecified unstable morphologies (AO/OTA 31-A2 vs 31-A3) were planned a priori to improve clinical interpretability. Analyses were primarily descriptive for this retrospective single-cohort study; however, exploratory comparisons between prespecified AO/OTA 31-A2 and 31-A3 subgroups were conducted to assess associations between fracture morphology and perioperative burden, union, complications, and functional outcome. Continuous variables were compared using Welch's t-test where appropriate; categorical variables were compared using Pearson's chi-square or Fisher's exact tests. Two-sided p-values are reported for transparency with α = 0.05.

## Results

Exploratory comparisons between the AO/OTA 31-A2 and 31-A3 subgroups were performed to enhance interpretability. Continuous variables were compared using an independent-sample Welch's t-test, and categorical variables were compared using Pearson's chi-square or Fisher's exact tests, as appropriate. All p-values are two-sided, and p < 0.05 was considered statistically significant. A total of 128 eligible patients underwent PFN for unstable intertrochanteric fractures. Baseline demographics were comparable between fracture subgroups (Table 1).

**Table 1:** Baseline demographic and clinical characteristics (n = 128) Data are mean ± SD or n (%). Welch's t-test was used for continuous variables, and Pearson's chi-square for categorical variables. Two-sided p < 0.05 was considered statistically significant. AO/OTA: AO Foundation/Orthopaedic Trauma Association; SD: standard deviation

Parameter	Overall (n = 128)	AO/OTA 31-A2 (n = 78)	AO/OTA 31-A3 (n = 50)	Test statistic	p-value
Age (years)	72.6 ± 9.4	71.8 ± 8.9	73.9 ± 10.1	t = -1.20	0.233
Age ≥60 years	101 (78.9%)	59 (75.6%)	42 (84.0%)	χ² = 1.28	0.258
Female sex	71 (55.5%)	42 (53.8%)	29 (58.0%)	χ² = 0.21	0.645
≥1 Medical comorbidity	86 (67.2%)	50 (64.1%)	36 (72.0%)	χ² = 0.86	0.353

Fracture morphology differed substantially, with higher frequencies of medial comminution, lateral wall compromise, and reverse-obliquity patterns in 31-A3 fractures (Table 2).

**Table 2:** Injury characteristics and fracture morphology (n = 128) Data are n (%). Pearson's chi-square was used, except where Fisher's exact test was required. Two-sided p < 0.05 was considered statistically significant.

Parameter	Overall (n = 128)	31-A2 (n = 78)	31-A3 (n = 50)	Test statistic	p-value
Low-energy fall	104 (81.3%)	61 (78.2%)	43 (86.0%)	χ² = 1.22	0.270
Road traffic accident	24 (18.7%)	17 (21.8%)	7 (14.0%)	χ² = 1.22	0.270
Medial comminution	92 (71.9%)	49 (62.8%)	43 (86.0%)	χ² = 8.10	0.004
Lateral wall compromise	68 (53.1%)	31 (39.7%)	37 (74.0%)	χ² = 14.36	<0.001
Reverse-obliquity pattern	41 (32.0%)	0 (0.0%)	41 (82.0%)	Fisher exact	<0.001

Specifically, 31-A3 fractures were associated with greater perioperative burden, including longer time to surgery, longer operative time, higher intraoperative blood loss, and more frequent open reduction. Because the fracture subgroup reflects greater structural instability and operative complexity, these findings were interpreted as associative. In adjusted analyses controlling for age, sex, and comorbidity burden, the direction of these associations remained consistent (Table 3).

**Table 3:** Operative and perioperative parameters (n = 128) Data are mean ± SD or n (%). Welch's t-test was used for continuous variables, and Pearson's chi-square for categorical variables. Two-sided p < 0.05 was considered statistically significant.

Parameter	Overall (n = 128)	31-A2 (n = 78)	31-A3 (n = 50)	Test statistic	p-value
Time to surgery (days)	3.2 ± 1.6	2.9 ± 1.4	3.7 ± 1.8	t = -2.67	0.009
Operative time (minutes)	62.4 ± 14.8	58.6 ± 12.9	68.3 ± 16.2	t = -3.57	<0.001
Intraoperative blood loss (mL)	265 ± 85	245 ± 72	295 ± 96	t = -3.16	0.002
Blood transfusion required	39 (30.5%)	19 (24.4%)	20 (40.0%)	χ² = 3.52	0.061
Open reduction required	18 (14.1%)	6 (7.7%)	12 (24.0%)	χ² = 6.70	0.010

Radiological union was achieved in 120 patients (93.8%), and time to union was longer in 31-A3 fractures (Table 4).

**Table 4:** Radiological outcomes and follow-up (n = 128) Data are mean ± SD or n (%). Welch's t-test was used for continuous variables. Pearson's chi-square or Fisher's exact test was used for categorical variables. Two-sided p < 0.05 was considered statistically significant. OR: odds ratio

Parameter	Overall (n = 128)	31-A2 (n = 78)	31-A3 (n = 50)	Test statistic	p-value
Radiological union	120 (93.8%)	75 (96.2%)	45 (90.0%)	χ² = 1.97	0.161
Time to union (weeks)	12.1 ± 2.9	11.4 ± 2.5	13.2 ± 3.2	t = -3.37	0.001
Delayed union	6 (4.7%)	2 (2.6%)	4 (8.0%)	OR = 3.30 (Fisher)	0.208
Non-union	2 (1.6%)	1 (1.3%)	1 (2.0%)	OR = 1.57 (Fisher)	1.000
Follow-up duration (months)	15.6 ± 4.9	15.1 ± 4.3	16.3 ± 5.4	t = -1.32	0.189

The mean MHHS was lower in 31-A3 fractures (84.8 ± 11.2) than in 31-A2 fractures by 4.6 points (p = 0.016) (Table 5). Although this average difference is modest, the distribution suggested a clinically relevant shift at the lower end, with a higher proportion of “Poor” outcomes in 31-A3 fractures (16.0% vs 3.8%), while the overall four-category comparison did not reach statistical significance (p = 0.064) (Table 5).

**Table 5:** Functional outcomes at final follow-up (Modified Harris Hip Score, n = 128) Data are mean ± SD or n (%). Welch's t-test was used for the mean MHHS. Pearson's chi-square was used for the four-category distribution comparison (single overall χ² and p-value shown). Two-sided p < 0.05 was considered statistically significant. The mean MHHS difference between groups was 4.6 points (31-A2 higher), with an approximate 95% CI of 0.9-8.3 (p = 0.016).

Parameter	Overall (n = 128)	31-A2 (n = 78)	31-A3 (n = 50)	Test statistic	p-value
MHHS (mean score)	87.6 ± 9.8	89.4 ± 8.7	84.8 ± 11.2	t = 2.47	0.016
Excellent (≥90)	62 (48.4%)	42 (53.8%)	20 (40.0%)	χ² = 7.25 (overall)	0.064 (overall)
Good (80–89)	41 (32.0%)	26 (33.3%)	15 (30.0%)	-	-
Fair (70–79)	14 (10.9%)	7 (9.0%)	7 (14.0%)	-	-
Poor (<70)	11 (8.6%)	3 (3.8%)	8 (16.0%)	-	-

Complication categories were not mutually exclusive, and some patients experienced more than one event. At the patient level, the proportion with any mechanical complication was higher in 31-A3 fractures than in 31-A2 fractures, while individual complication types showed similar directionality, but most comparisons did not reach statistical significance, likely due to limited event counts (Table 6).

**Table 6:** Postoperative complication profile (n = 128) Data are n (%). Pearson's chi-square was used, except where Fisher's exact test was required, in which case, the odds ratio (OR) is reported. Two-sided p < 0.05 was considered statistically significant.

Parameter	Overall (n = 128)	31-A2 (n = 78)	31-A3 (n = 50)	Test statistic	p-value
Z-effect phenomenon	5 (3.9%)	1 (1.3%)	4 (8.0%)	OR = 6.70 (Fisher)	0.076
Screw cutout	7 (5.5%)	2 (2.6%)	5 (10.0%)	OR = 4.22 (Fisher)	0.109
Varus collapse (>5 degrees)	9 (7.0%)	3 (3.8%)	6 (12.0%)	χ² = 3.10	0.078
Surgical site infection	8 (6.3%)	5 (6.4%)	3 (6.0%)	χ² = 0.01	0.925
Limb length discrepancy (>1 cm)	10 (7.8%)	4 (5.1%)	6 (12.0%)	χ² = 2.00	0.158

## Discussion

In this rural retrospective study of unstable intertrochanteric fractures treated with PFN, union, complication profile, and functional outcomes provide descriptive, real-world benchmarks for this implant within the local care pathway. Given the absence of a comparator implant or non-operative control, these findings should be interpreted as associative and descriptive rather than confirmatory of implant effectiveness while still highlighting a consistent severity gradient in which AO/OTA 31-A3 patterns were associated with greater operative burden, slower union, and lower functional scores [1,11]. The internal gradient across fracture types was consistent: compared with 31-A2, 31-A3 fractures had longer operative time, higher blood loss and transfusion requirement, greater need for open reduction, slower union, lower MHHS, and higher rates of Z-effect, screw cutout, and varus collapse. These findings are biomechanically plausible because reverse-oblique and multifragmentary patterns commonly combine posteromedial loss with lateral wall compromise, increasing varus moments and head-neck stresses that promote migration and collapse if reduction and fixation are suboptimal [1,14].

The overall union and functional profile aligns with published PFN series reporting high healing rates with predominantly good-to-excellent short-term hip function, alongside persistent implant-related and functional sequelae. Chopra et al. documented union in nearly all cases with a complication spectrum, including Z-effect or reverse Z-effect, stiffness, shortening, and hardware-related symptoms [3], and Gadegone et al. similarly reported good-to-excellent outcomes in most patients but noted varus collapse, cutout, Z-effect variants, infection, and shortening [2]. MHHS-based outcomes in the present study are directionally consistent with those of James et al., where most patients achieved excellent-to-good function at six months, yet a minority remained fair or poor, reflecting the combined influence of fracture instability, comorbidity burden, and rehabilitation variability [4]. Importantly, functional recovery after PFN can remain incomplete even when radiographic union is achieved, because gait studies demonstrate persistent deficits versus healthy controls at six months, particularly in single-limb support and propulsion phases that are sensitive to abductor mechanics and overall physiologic reserve [12]. This helps explain why a subset in the current study group had a poor MHHS despite a high union rate and why the outcomes were worse in the more unstable 31-A3 group.

The mechanical complication profile in this cohort is best interpreted using known modifiable determinants, especially reduction quality and implant positioning. Radiographic predictor studies show that unsatisfactory reduction is a dominant driver of cutout after cephalomedullary fixation, sometimes exceeding the predictive value of classical TAD in multivariable analysis [15]. Other cohorts demonstrate that a positioning threshold effect may still matter, with higher cutout risk when TAD is 25 mm or more, particularly when reduction quality is inferior [10]. Although reduction quality and implant position metrics (e.g., Baumgaertner grading, TAD/CalTAD) are established determinants of cutout and varus collapse, these variables were not available in a complete, uniformly measurable form across the retrospective dataset and were therefore not analyzed as explanatory covariates. Accordingly, the higher cutout and varus collapse rates in 31-A3 fractures, and the greater need for open reduction, should be interpreted as associations reflecting greater fracture instability and operative complexity rather than as mechanistic effects attributable to reduction quality or screw position within this cohort [11,14]. Lateral wall behavior is also relevant in A3 patterns, because displaced lateral wall fragments may remodel postoperatively, but the initial configuration can still affect sliding, telescoping, and collapse risk [16].

Where this cohort differs from some reports, plausible explanations include case-mix and definitional heterogeneity. The observed mean union time appears earlier than in some instability-stratified series, which may reflect differences in union definitions, follow-up imaging intervals, and fracture subtype composition [2,4]. Conversely, the concentration of mechanical complications in 31-A3 fractures may be amplified by the very high prevalence of reverse obliquity within that subgroup and the coexistence of medial comminution and lateral wall compromise, both of which predispose to varus and migration when cortical support is not re-established [11,14]. Implant configuration may also contribute to highly unstable patterns. In reverse-oblique fractures, retrospective evidence suggests that certain nail configurations and nail lengths may influence cutout risk, although confounding by implant selection and surgeon factors remains important [8]. Biomechanical modeling in an AO/OTA 31-A3.3 construct demonstrated greater varus-related angular changes and construct-related complications with short nails compared with mid and long nails, supporting consideration of longer constructs for selected highly unstable morphologies when feasible [17].

Clinically, these findings support PFN as a practical fixation strategy for unstable intertrochanteric fractures in rural systems, provided that attention is intensified for 31-A3 patterns. Preoperative characterization of medial support, lateral wall integrity, and reverse obliquity should directly inform intraoperative tactics aimed at avoiding varus, restoring cortical continuity where possible, and achieving stable proximal fixation, because the literature repeatedly links these steps to lower failure rates [1,10,15]. In unstable fractures with deficient lateral walls, augmentation strategies may be relevant. Randomized evidence indicates that adding a lateral wall plate to PFN can improve neck-shaft angle maintenance and reduce varus collapse with better short-term hip function [18], and screw augmentation of PFN has been associated with improved scores and fewer collapse-related problems in unstable A2 fractures. Patient factors also matter: worse postoperative function with severe osteoporosis has been documented after PFN [9], and higher comorbidity burden has been implicated in revision risk in large retrospective analyses [19], supporting risk-stratified rehabilitation planning and follow-up intensity. Rare but serious complications must remain on the radar because distal interlocking can injure femoral vessels and present as thigh pain, swelling, and unexplained anemia, warranting early CT angiography when suspected [5]. For selected very elderly unstable fractures where immediate full weight-bearing is critical, arthroplasty can accelerate early mobilization but commonly increases blood loss and transfusion exposure, with many outcomes converging later, so decisions should be individualized based on physiologic reserve and resource realities [7,11].

The strengths of this study include a focused evaluation of unstable AO or OTA 31-A2 and 31-A3 intertrochanteric fractures within a clearly defined rural care pathway, the use of standardized fracture classification with blinded radiographic review, consistent operative principles during the study period, and a clinically meaningful follow-up duration sufficient to capture union, mechanical complications, and functional recovery. Reporting morphology-stratified outcomes provides practical benchmarks for similar resource-constrained settings and enhances interpretability beyond the pooled hip-fracture study group.

The limitations include the retrospective single-center design with potential selection bias related to documentation quality and follow-up completeness, and the absence of a comparator implant or non-operative control, which restricts conclusions to descriptive PFN outcomes rather than comparative effectiveness. Important confounders such as pre-injury mobility, baseline functional status, objective bone-quality assessment, and structured rehabilitation exposure were not uniformly captured. Although radiographic parameters, including reduction quality and implant position, were reviewed, they were not consistently available in a form suitable for explanatory modeling, limiting mechanistic inference regarding cutout and varus collapse. Complication analyses were constrained by low event counts, possible under-ascertainment of events managed outside the institution, and non-mutually exclusive complication categories. Subgroup comparisons between 31-A2 and 31-A3 patterns remain exploratory and may retain residual confounding related to fracture complexity and operative decision-making.

## Conclusions

PFN was associated with high rates of fracture healing and generally satisfactory hip function in unstable intertrochanteric fractures managed in a rural care pathway. Postoperative mechanical complications and wound-related events occurred and should be interpreted as expected risks in this injury spectrum rather than as evidence for or against implant superiority in the absence of a comparator group. Outcomes were consistently less favorable in the more unstable AO or OTA 31-A3 patterns than in 31-A2 fractures, suggesting that greater fracture instability is associated with higher mechanical risk and slower functional recovery. These findings support careful preoperative fracture characterization, meticulous reduction, and stable implant positioning with a low threshold for structured follow-up and early detection of collapse or migration events, particularly in highly unstable patterns.

## References

[1] Mavrogenis AF, Panagopoulos GN, Megaloikonomos PD (2016). Complications after hip nailing for fractures. Orthopedics.

[2] Gadegone WM, Salphale YS (2010). Short proximal femoral nail fixation for trochanteric fractures. J Orthop Surg (Hong Kong).

[3] Chopra BL, Kumar K, Khajotia BL, Bhambu R, Bhatiwal S, Shekhawat V (2017). Proximal femoral nail outcome and complications: a prospective study of 125 cases of proximal femoral fractures. Int J Res Orthop.

[4] James B, Prasath R, Vijayakumaran Vijayakumaran (2017). Functional outcome of proximal femoral nailing in inter trochanteric fractures of femur: a prospective study. Int J Orthop Sci.

[5] Yoon HK, Oh HC, Park J, Oyunbat C, Kim T (2016). Rupture of the deep femoral artery during proximal femoral nailing following an intertrochanteric fracture: a case report. Hip Pelvis.

[6] Musa AH, Mohamed MS, KhalafAllah HG (2025). Dynamic hip screw versus proximal femoral nailing in stable intertrochanteric fractures: a systematic review of efficacy and outcomes. BMC Musculoskelet Disord.

[7] Zhang H, Zeng X, Zhang N (2017). INTERTAN nail versus proximal femoral nail antirotation-Asia for intertrochanteric femur fractures in elderly patients with primary osteoporosis. J Int Med Res.

[8] Elbaz E, Morgan S, Factor S (2023). Reduced cutout for reverse oblique intertrochanteric hip fractures treated with trochanteric fixation advanced (TFN-A) nail compared to the short gamma-3 nail. SICOT J.

[9] Akan K, Cift H, Ozkan K, Eceviz E, Tasyikan L, Eren A (2011). Effect of osteoporosis on clinical outcomes in intertrochanteric hip fractures treated with a proximal femoral nail. J Int Med Res.

[10] Donadono C, Tigani D, Assenza A, Censoni D, Pesce F, Melucci G (2025). Clinical outcomes in the treatment of pertrochanteric femur fractures: a retrospective cohort study. J Pers Med.

[11] Song H, Hu SJ, Du SC, Xiong WF, Chang SM (2021). Sub-classification of AO/OTA-2018 pertrochanteric fractures is associated with clinical outcomes after fixation of intramedullary nails. Geriatr Orthop Surg Rehabil.

[12] Sivakumar A, Rickman M, Thewlis D (2023). Gait biomechanics after proximal femoral nailing of intertrochanteric fractures. J Orthop Res.

[13] Lakhotia D, Agrawal U (2023). Functional outcome of uncemented total hip replacement in low socioeconomic group using modified Harris hip score: a prospective midterm follow-up study. Cureus.

[14] Hoffmann MF, Khoriaty JD, Sietsema DL, Jones CB (2019). Outcome of intramedullary nailing treatment for intertrochanteric femoral fractures. J Orthop Surg Res.

[15] Raghuraman R, Kam JW, Chua DT (2019). Predictors of failure following fixation of intertrochanteric fractures with proximal femoral nail antirotation. Singapore Med J.

[16] Kim Y, Bahk WJ, Yoon YC, Cho JW, Shon WY, Oh CW, Oh JK (2015). Radiologic healing of lateral femoral wall fragments after intramedullary nail fixation for A3.3 intertrochanteric fractures. Arch Orthop Trauma Surg.

[17] Ando J, Takahashi T, Matsumura T, Takeshita K (2024). Biomechanical comparison of short-, mid-, and long-length proximal femoral nails for femoral intertrochanteric fracture (AO/OTA 31A3.3) fixation. Geriatr Orthop Surg Rehabil.

[18] Lingayat MB, Agiwal Y, V V (2023). Comparative analysis of the clinical, functional and radiological outcomes of proximal femoral nail alone verses proximal femoral nail along with lateral wall plating in management of unstable intertrochanteric femur fracture, AO/OTA, type 31A2 fractures with deficient lateral wall. Int J Res Orthop.

[19] Duman E, Torun Ö, Girgin AB, Özçelik MA, Acar A, Çevik HB (2025). Evaluation of risk factors for revision surgery after proximal femoral nailing for intertrochanteric fractures. Medicina (Kaunas).

